# Microbiome composition and autochthonous probiotics from contrasting probiosis/dysbiosis states in cobia (*Rachycentron canadum*) fish epitheliocystis

**DOI:** 10.1099/acmi.0.000405

**Published:** 2022-08-10

**Authors:** Marcela Villegas-Plazas, Luisa Villamil, María Angélica Martínez-Silva, Tatiana González-Jiménez, Marcela Salazar, Linda Güiza, Mabel Mendoza, Howard Junca

**Affiliations:** ^1^​ RG Microbial Ecology: Metabolism, Genomics & Evolutions, Div. Ecogenomics & Holobionts, Microbiomas Foundation, Chia, Colombia; ^2^​ Universidad Jorge Tadeo Lozano, Sede Santa Marta, Colombia; ^3^​ Programa de Doctorado en Biociencias, Facultad de Ingeniería, Universidad de la Sabana, Chía, Colombia; ^4^​ Université du Québec à Rimouski, Institute des Sciences de la Mer à Rimouski, Québec, Canada; ^5^​ Corporación CorpoGen, Bogotá, Colombia; ^6^​ Benchmark Genetics Colombia, Punta Canoas, Cartagena, Colombia; ^7^​ Centro de Investigación de la Acuicultura en Colombia, Ceniacua, Cartagena, Colombia

**Keywords:** cobia, dysbiosis, epitheliocystis, fish, *Mesobacillus*, microbiome, probiosis

## Abstract

Microbiome components and bacterial isolates related to healthy and epitheliocystis states in aquaculture cycles of cobia fish were studied. We detected well-defined 16S rRNA amplicon gene sequence variants showing differential abundance in healthy or diseased cycles. Isolation trials were performed, and experimental tests were used to determine probiotic potential of the bacterial strains obtained from water, tissues or live food used in this aquaculture model. The taxonomic affiliation of these strains was cross-compared against microbiome components, finding that some of them had close or identical affiliation to the abundant types found in healthy cycles. Strains belonging to the groups already identified as predominant by culture-independent means were screened as potential probiotics based on desirable activities such as antagonism and antibiosis against marine pathogenic bacteria, quorum quenching, bile acid resistance, antibiotic sensitivity and enzymatic activities for improved nutrient digestion. We have also found that in the tracking of microbiome composition across different developmental stages of cobia, healthy cycles exhibited a consistent high relative abundance of a *Mesobacillus* sp., while in the diseased cycle the emergence of a *Vibrio* sp. was observed. Our study suggests that epithelocystis in cobia is associated with a displacement of a symbiotic microbiome community linked to the increase frequency of *Vibrio* species.

## Data availability

16S rRNA gene sequences are available in the GenBank NCBI database under the ID PRJNA796496, accession numbers SAMN24867264–SAMN24867319.

## Introduction

Aquaculture is one of the most promising responses to the increasing global demand for animal proteins. It is largely dependent on guaranteeing suitable and sustainable conditions for the growth of healthy marine organisms. Cobia (*Rachycentron canadum*, L.) culture has the potential to become an important cultured species due to its advantageous features for commercial production, such as high growth rates, adapted aquaculture performance and fillet quality [1]. Nevertheless, after 2014, experimental and industrial cobia culture experienced frequent disease incidents, vibriosis being the one most commonly reported [2]. During the last decade, cobia production in the South Caribbean Sea of the Colombian Northwestern shore faced three severe mortality outbreaks caused by epitheliocystis, a common disease characterized by the onset of cysts in the gills of the cobia larvae, reducing survival rates to an average of 5 % [3, 4]. This caused severe economic losses, and additionally hampered, together with extreme climatic events, continuation of this productive activity in the country. Previous studies have suggested that cobia epitheliocystis is caused by a close relative of the bacterium *Endozoicomonas elysicola* [4], which has been reported within the normal microbiota of corals and bivalves [5].

It is well known that the indigenous fish microbiota plays a significant role not only in the growth and development of the host, but also in the protection and immune response against pathogens [6–8]. However, there is no published information regarding microbial communities associated with healthy and diseased cobia fish gills or the probiotic potential of the bacteria commonly associated with the culture and host.

The use of microorganisms with probiotic potential in aquaculture to prevent infectious disease episodes is increasing [9, 10]. There are multiple studies reporting the use of *Bacillaceae* strains as an alternative approach to substitute antimicrobial compounds in cobia culture [11, 12], and studies have also shown that cobia itself is a promising resource to isolate bacteria with probiosis potential [13]. Although effective, the use of commercial probiotics is still limited due to high costs and the poor *in situ* performance of allochthonous strains. Therefore, the generation of microbiome-guided autochthonous probiotic strain collections, such as those types positively selected or associated with a healthy status on microbiome assessments, could provide ecological and sustainability advantages towards a more efficient and appropriate protection regime. Here we studied the cobia aquaculture system as a model for the isolation of autochthonous bacterial probiotics, defining its healthy microbiome components that may help to improve fish health and productivity.

**Fig. 1.**
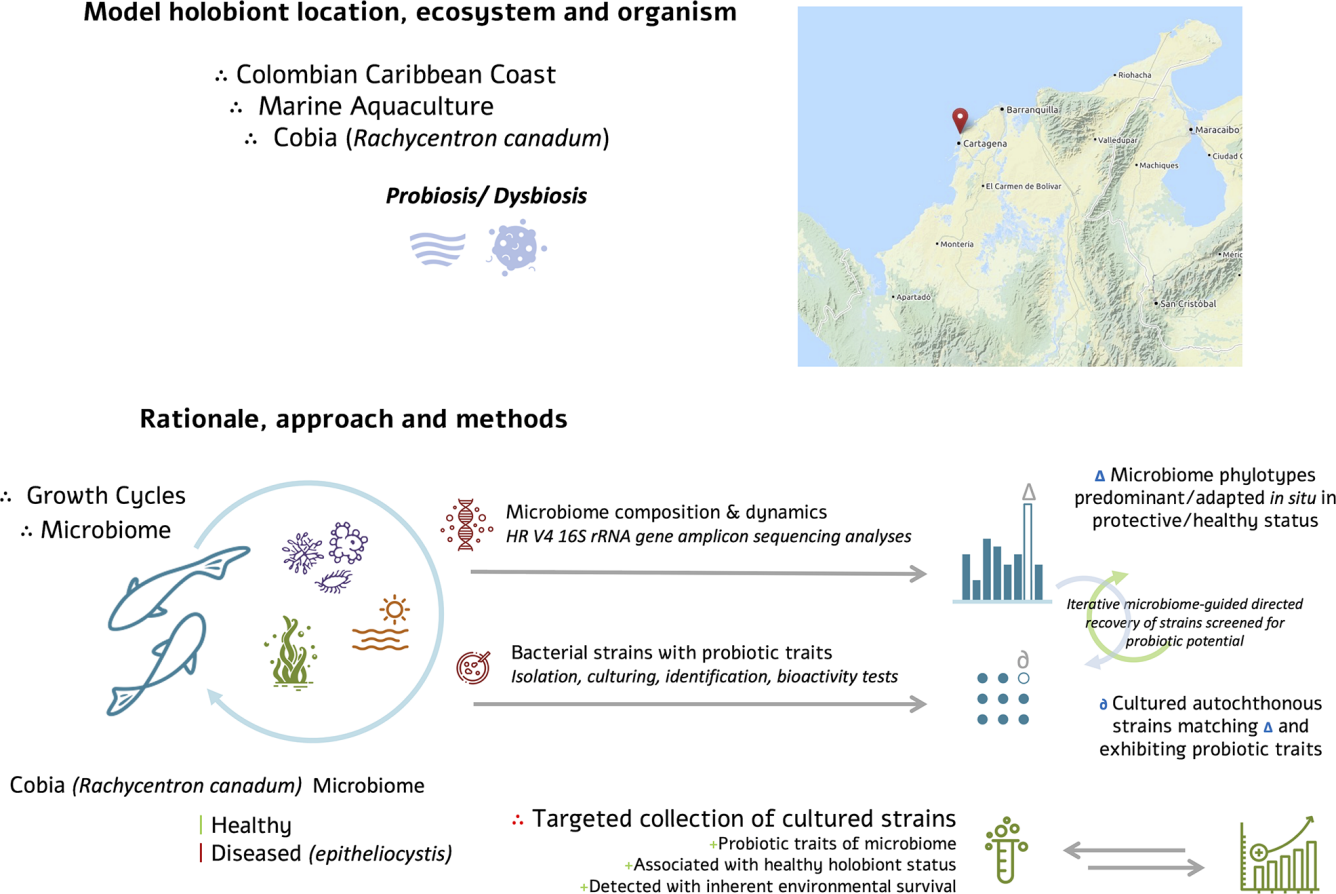
Rationale for microbiome-based guided autochthonous probiotic isolation in cobia as a model holobiont. (Icon sources under CC-BY-SA 2.0. Map source is OpenStreetMap under distribution licence ODbL 1.0.)

We report the diversity of microbial communities associated with the gills in healthy larvae and larvae with epitheliocystis symptoms. Furthermore, we isolated bacteria with similar identities to those associated with healthy cobia (*Rachycentron canadum*), and assessed the probiotic potential with promising results (Fig. 1).

## Methods

### Sample collection for microbiome analyses

Complete growth cycles of cobia from egg hatching and larvae were established in experimental aquaculture aquaria in tanks located at the Colombian Caribbean coast and followed for 2 years. Sequential samples were collected, in one case tracking those that represented a consistent healthy cycle, with no signs of disease, and in another those that presented clear outbreaks of epitheliocystis disease. The experiments started in 2012 and finished at the end of 2014. A total of 56 larvae samples were selected; 18 corresponded to a healthy cycle (samples labelled with prefix ‘A’), while 38 corresponded to cycles of cyst larvae maintained and grown in two different tanks and times [samples coded with prefix ‘B’ (tank 2) and those labelled with prefix ‘C’ (tank 3) are those where disease outbreaks were detected]. After collection, all samples were preserved in ethanol at −20 °C.

### DNA extraction

Total DNA was extracted from 0.25 g of cobia gill tissue using a combined version of two different methodologies previously described for the extraction of DNA from fishes [14] and natural environments [15]. Briefly, the samples were first crushed and mixed using a Bead Beater with the same volume of silica beads and 400 µl of Extraction Buffer (10 mM Tris-HCl, pH 8.0; 20 mM EDTA, pH 8.0; 2 % SDS; 6 M urea) [14]. In total, 20 µl of 10 mg ml^−1^ Proteinase K was added followed by an overnight incubation at 55 °C. A 0.1 volume of 5 M NaCl was added and gently mixed by inversion. An equal volume of phenol–chloroform–isoamyl alcohol (25 : 24 : 1, pH 8) was added, vortexed briefly, and the aqueous phase was separated by centrifuging for 10 min (13 000 r.p.m. at 4 °C) and mixed with an equal volume of chloroform–isoamyl alcohol (24 : 1) followed by 10 min of centrifugation (13 000 r.p.m. at 4 °C). Total DNA was precipitated from the extracted aqueous layer adding an equivalent of 0.1× supernatant volume of 3 M sodium acetate and 0.6× supernatant volume of isopropanol. Samples were incubated overnight at −20 °C followed by 30 min of centrifugation (13 000 r.p.m. at 4 °C). The resulting DNA pellets were then washed in ice-cold 70 % (v/v) ethanol, dried and re-suspended in 30 µl of TE Buffer. DNA was quantified using a Nanodrop ND-1000 (Thermo Scientific).

### 16S rRNA gene amplification and sequencing

PCR amplicon libraries of the V4 region of the 16S rRNA genes were prepared to determine the bacterial and archaeal compositions in the samples. To obtain these amplicons from metagenomic DNA of cobia gills, the primers 515F [5′-GTGCCAGCMGCCGCGGTAA- 3′] and 806R [5′-GGACTACHVGGGTWTCTAAT-3′] were used [16]. Each 25 µl reaction comprised 1× buffer, 0.3 mM dNTPs, 0.5 µM forward primer, 0.5 µM reverse primer, 0.04 U µl^–1^ Accuzyme polymerase (Bioline) and 1 ng of template DNA. The PCR cycle conditions comprised initial denaturation at 94 °C for 3 min followed by 35 cycles of denaturation at 94°C for 45 s, primer annealing at 50 °C for 60 s and extension at 72 °C for 90 s. This was followed by a final extension at 72 °C for 10 min [17]. Sequencing was done using the Miseq Illumina platform (Baylor College of Medicine, Houston, TX, USA).

### Diversity analysis

All sequence analyses were performed using QIIME 1.9.1 [18]. Initially, the set of Illumina reads was filtered and split according to the barcodes. Chimeric sequences were identified, extracted and excluded from the datasets by Usearch 6.1. A multi-step open-reference operational taxonomic unit (OTU) picking workflow was performed within the QIIME system. Using this methodology, OTUs were picked assigning reads to species groups based on 97 % sequence similarity. This workflow combined the PyNAST alignment [17] against the silva core set to make the OTUs assignment and to discard chimaric sequences. In the next step, a single representative sequence of each OTU was again realigned using PyNAST to build a phylogenetic tree using FastTree. Alpha and beta diversity analyses were performed with both platforms, QIIME and the Phyloseq R package [19].

### Correlation and statistical analysis

Multivariate data analysis was used to explore the patterns in the global microbial community with respect to sickness condition, each sample was compared to each other sample via ordinated principal coordinate analysis (PCoA) following construction of a sample–similarity matrix using either the weighted unifrac distance or the global microbial profiles [20]. Significant differences between the relative abundance of each OTU across the sickness condition were evaluated using one-way ANOVA. OTUs were considered significantly different if the p-value fallsat *P*<0.001. These analyses were performed in R. The percentage of each of these significantly different OTUs in the both datasets, sick and healthy, was then calculated, and those with differences >5 % between the two datasets were selected as the most influential OTUs for the sickness condition. Spearman’s rank correlation was performed to determine the co-occurrence between all OTUs [21]; Spearman’s correlation coefficient was calculated using the stats and lineup packages of R.

### Potential probiotic isolation and biochemical characterization

Bacteria were isolated from samples collected from cycles of cobia larval cultures, from subsamples of culture water, gills and larval intestines, as well as *Artemia* and rotifers used for feeding. Serial dilutions of samples were cultured in Marine Agar (BD Difco) (https://www.dsmz.de/microorganisms/medium/pdf/DSMZ_Medium123.pdf) and Man Rogosa Sharpe agar (MRS; Oxoid) (https://www.dsmz.de/microorganisms/medium/pdf/DSMZ_Medium11.pdf) at room temperature (20 °C) and 36 °C for 48–72 h under aerobic conditions. The colonies were isolated and stored in the corresponding medium with 15 % glycerol for storage at −20 or −80 °C [22]. In addition, the morphology, Gram character and glucose fermentation of all the isolates were studied, and a more detailed morphological and biochemical characterization of the selected isolates was carried out.

### Evaluation of antimicrobial activity of extracellular products (ECPs)

Bacterial ECPs were obtained according to previously described methodologies [23]. Briefly, each bacterium was seeded in 10 ml of TSB (https://www.dsmz.de/microorganisms/medium/pdf/DSMZ_Medium545.pdf) with 1 % NaCl or MRS for 24 h at room temperature (20 °C). Three cycles of centrifugation were performed at 500 *g* for 15 min, taking the supernatant each time; the final supernatant was filtered at 0.45 µm and pH was adjusted to 7 and aliquoted for future analysis at −80 °C. The antimicrobial activity assay was carried out in a sterile 96-well plate, placing 50 µl of the obtained ECPs or medium in the case of controls and 50 µl of the pathogenic bacteria at 1×10^6^ c.f.u. ml^−1^. Bacterial concentration was determined based on the optical density (OD) at 600 nm (Modulus II microplate multimode reader) and verified by plate counting. After 24 h of incubation at room temperature (20 °C), the absorbance was measured at 600 nm. The result was expressed as a percentage of inhibition, taking into account that the values obtained from the control of each of the pathogenic bacteria studied without ECPs of the possible probiotics corresponds to 100 % growth. The pathogenic bacteria used to determine antimicrobial activity were *Aeromonas hydrophila *, *Vibrio alginolyticus *, *Edwardsiella tarda* and *Streptococcus agalactiae *.

### Quorum quenching (QQ)

Assays were carried out according to the previously proposed methodology [24]. Sterile filter paper discs with 10 µl of ECPs or active bacteria isolated from cobia culture were placed on a culture of *Chromobacterium violaceum* previously plated on Casein peptone Soybean Flour Peptone Agar (CASO agar; Sigma-Aldrich) (https://www.dsmz.de/microorganisms/medium/pdf/DSMZ_Medium220.pdf). Plates were incubated at 30 °C for 24 h and the presence of halos of inhibition pigment production was measured. The result was expressed in millimetres and was considered positive for QQ or quorum sensing (QS) disruption. According to the results obtained in antimicrobial activity and QS inhibition tests, ten isolates were selected for molecular characterization. Their ability to inhibit biofilm formation, antibiotic susceptibility, enzyme activity and bile salt tolerance was also evaluated.

### Biofilm inhibition

In a 96-well plate, 180 µl of the biofilm-forming bacteria *Photobacterium* sp. and *Bacillus pumilus* and 20 µl of the ECPs of potential probiotic bacteria were placed in triplicate. As a positive control, 180 µl of culture of the biofilm-forming bacteria (1×10^6^ c.f.u. ml^−1^) and 20 µl of TSB +1 % NaCl were used. The plate was incubated at 37 °C for 24 h. Subsequently the plate was emptied and washed three times with distilled water. After 10 min of drying, plates were fixed with 250 µl of 90 % methanol for 15 min and dry for 10 min, and 200 µl of 1 % crystal violet was added and washed three times with distilled water. After drying, 250 µl of 95 % ethanol wasadded and, finally, the absorbance was measured at 600 nm.

### Antibiotic sensitivity

Bacteria were inoculated into 10 ml of marine broth (BD Difco) and MRS broth (Oxoid) for 24 h at 20 °C. Each bacterium was adjusted to 1×10^6^ c.f.u. ml^−1^ and plated on Muller Hinton agar (Sigma-Aldrich). Discs with antibiotics (Oxoid) at different concentrations (µg per disc) were used: tetracycline (30), chloramphenicol (30), oxolinic acid (2), nitrofurantoin (300), ciprofloxacin (5), trimethoprim sulfamethoxazole (25) and streptomycin (10). After the incubation period, the presence or absence of an inhibition halo was observed.

### Enzymatic activity

Enzymatic activity was studied using API ZYM (bioMérieu). The bacteria were cultured on TSA (Sigma) (https://www.dsmz.de/microorganisms/medium/pdf/DSMZ_Medium535.pdf) and, after 24 h, bacteria were resuspended in in 2 ml of API Suspension Medium (bioMérieux) and adjusted to a turbidity of 5–6 on the McFarland scale (approx. 1.5–1.9×10^9^ c.f.u. ml^−1^). Aliquots of 65 µl of the suspensions were added to each of the 20 reaction cupules in the API ZYM strip. The strips were incubated at 20 °C for 4.5 h and the reactions were developed by the addition of one drop each of the API ZYM reagents A and B. Enzymatic activities were graded from 0 to 5, and converted to nanomoles as indicated by the manufacturer’s instructions.

### Bile salt tolerance

This was determined by modifying the technique of Erkkilä and Petäjä [25]. Briefly, bacterial suspensions (1×10^6^ c.f.u. ml^−1^) were inoculated into 7 ml TSB broth+1 % NaCl with bile salts (5, 3 and 1 %) (Sigma). Subsequently, they were incubated at 20 °C for 24 h and the absorbance (600 nm) was determined. Data were reported as a percentage growth ±se, using the OD value of the controls as 100 % bacterial growth.

### Molecular identification

Genomic DNA was obtained with the Maxwell automated system (Promega). For amplification of the 16S rRNA hypervariable regions V1–V8, the primers and amplification conditions used were followed Chakravorty *et al*. [26]. The products were visualized by agarose gel electrophoresis (1 %), and bands were purified and sequenced at both ends. The sequences were analysed by Blast in the GenBank database and the Ribosomal database Project (RDP). For assignment of the closest genus and species with the most similar sequence reported in the database, a minimum of 500–550 bases was considered, with a percentage of ambiguity <1 %. For identification of the closest species reported, we used the criterion of similarity of >99.5 % to the reference strain.

### Data analysis

Descriptive statistics were performed by expressing the results as mean±se using Microsoft Excel 2010. To determine significant differences between treatments, a comparison was made via the Kruskall–Wallis test (Statgraphics Centurion XV.I). Differences were determined to be statistically significant at *P*<0.05.

## Results

### Microbiome composition of cobia aquaculture in cycles with and without epitheliocystis

#### Bacterial alpha and beta diversity measures exhibit distinct well-defined patterns between health and disease status

Three complete growth cycles of cobia from egg hatching and larvae were used as experimental subjects in this study. One represented a completely healthy cycle, while in the other two an outbreak of epitheliocystis disease was detected. For characterization of the microbial communities associated with them, a total of 856 850 sequences of 250 nt on average were obtained from the Illumina-MiSeq platform. Following quality filtering steps and removal of chimeric sequences, 406 753 reads were clustered into OTUs at 97 % similarity. In total, 1727 OTUs were recovered from all the samples and were affiliated with 18 described bacteria phyla, four candidate phyla and three *Archaea* linages. In all communities associated with both sick and healthy individuals, taxa from the phyla *Proteobacteria* and *Firmicutes* comprised about 50 % of the community, while *Bacteroidetes* and *Cyanobacteria* were detected above 1 % abundance.

**Fig. 2.**
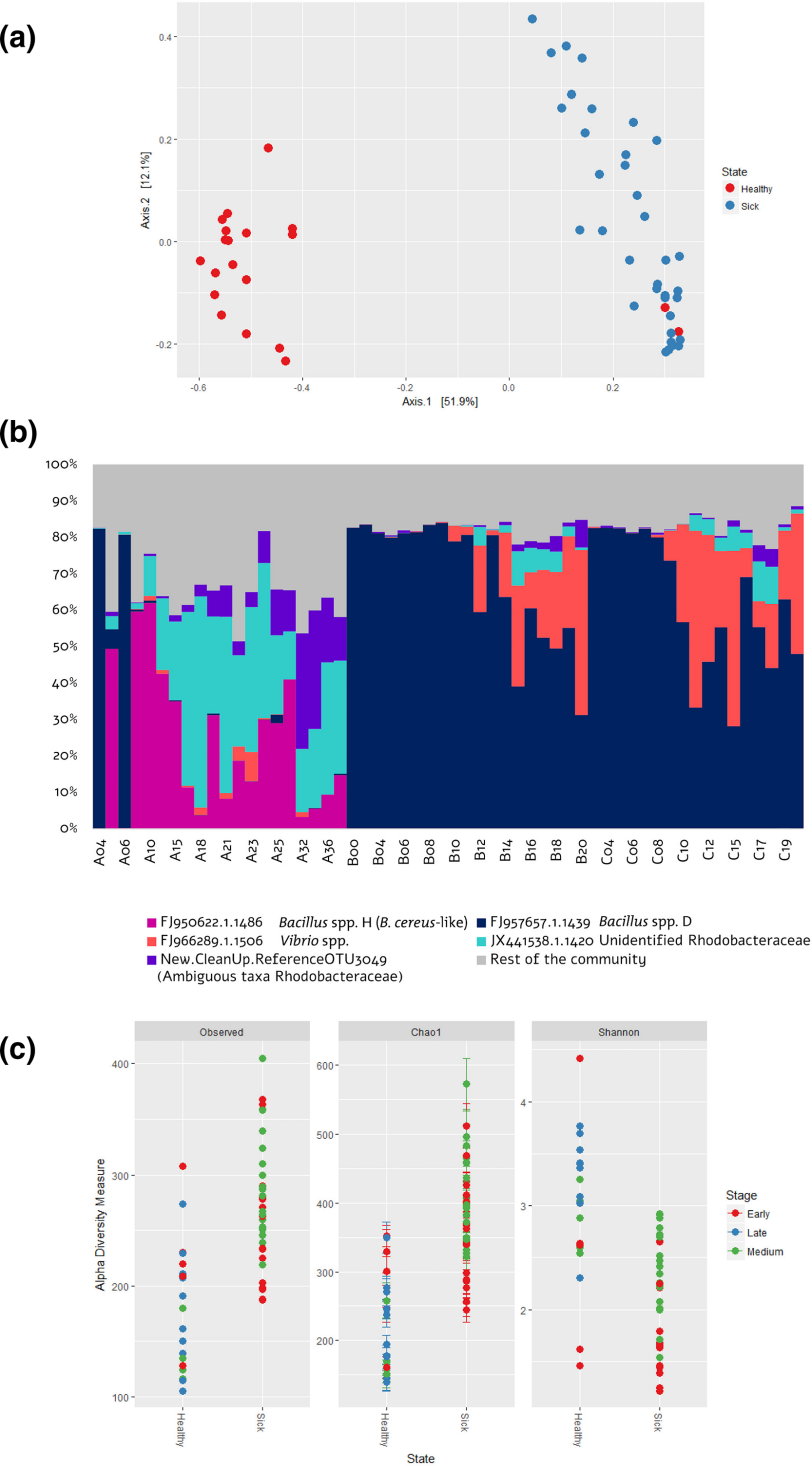
Microbiome taxonomy of cobia under healthy and epitheliocystis diseased cycles showing distinct compositional and diversity patterns, indicating the potential use of this information for guided probiotics isolation. (**a**) Ordination of the global microbial assemblages using PCoA. (**b**) Relative frequencies of the five selected OTUs as those with the most influence on the sickness condition, compared with the rest of the communities, inferred from 16S amplicon Illumina reads with an average of 7562 sequences per specimen. A, healthy cycle; B, sick cycle from tank 2; C, sick cycle from tank 3. The numbers of each sample indicate the day post-hatching (dph) of larvae sampled. (**c**) Species richness and alpha-diversity of all samples, differentiated according to sickness condition cycle and stage of the specimens collected.

Rarefaction analysis showed that the majority of members of the associated community for each sample were described: Chao1 estimator values suggested good agreement with the levels of coverage, showing high values >95 %. Species richness (observed species and Chao1) and alpha-diversity (Shannon) revealed variability between communities associated with healthy and diseased larvae (Fig. 2); richness was higher in those communities of specimens with symptoms of epitheliocystis. Ordination of the 56 samples via PCoA, which was performed following the use of the Bray–Curtis distance metric, also revealed a clear shift with respect to sickness on the PCO1 axis (Fig. 2a).

*Correlation between the structure of microbial communities and the health/disease status of larvae reveal specific bacterial types linked to each condition*.

A more detailed observation of the components of these communities showing significant differences with respect to the appearance of epitheliocystis symptoms was confirmed by one-way ANOVA, through which we identified 146 OTUs with highly significant difference (*P*<0.001), most affiliated to the families *Bacillaceae *, *Rhodobacteraceae* and *Vibrionaceae* (Table 1).

**Table 1.** Taxonomy and number of OTUs significantly different with respect to sickness condition

Family	No. of OTUs
* Flammeovirgaceae *	3
* Cryomorphaceae *	1
* Flavobacteriaceae *	7
* Parachlamydiaceae *	1
* Bacillaceae *	83
* Aerococcaceae *	1
* Carnobacteriaceae *	1
* Streptococcaceae *	1
* Peptostreptococcaceae *	1
* Hyphomonadaceae *	2
* Rhodobacteraceae *	24
*LWSR-14*	1
* Erythrobacteraceae *	1
* Bdellovibrionaceae *	2
* Aeromonadaceae *	1
* Alteromonadaceae *	1
* Cellvibrionaceae *	1
* Enterobacteriaceae *	2
* Pasteurellaceae *	1
* Pseudomonadaceae *	2
* Vibrionaceae *	9

Among these 146 OTUs, five were particularly notable: their relative abundances exhibited the largest contrast between healthy and diseased cohorts, presenting the greatest differences (5 –40 %); they accounted for >60 % of the total community associated with healthy larvae and >80 % of the total community associated with sick larvae. Fig. 2b shows that the predominant OTUs in healthy larvae were from the genus *Bacillus* and two unidentified genera within the family *Rhodobacteraceae *, while in the communities associated with epitheliocystis symptoms the predominant OTUs were in the genus *Vibrio* and a *Bacillus* sp. ecotype clearly distinct to the one found in the healthy cycle.

Spearman’s rank correlation between all OTUs showed that those predominant in sick larvae (FJ957657.1.1439 and FJ966289.1.1506 in Fig. 2c) did not show a robust co-occurrence with any other OTU from the whole dataset; while OTUs predominant in healthy larvae (FJ950622.1.1486, JX441538.1.1420 and New.CleanUp.ReferenceOTU3049 in Fig. 2) did show a valid co-occurrence with several OTUs, but all of them with the same taxonomy and were poorly represented (<20 sequences). No robust negative associations (Spearman’s coefficient close to −1) between characteristic OTUs of sick larvae and the rest of the community were found.

### Bacterial isolates from cobia tissues, feeding sources and aquaria waters: identity and relevant activities

*Taxonomic affiliations of the bacterial isolates from cobia belong to potential probiotic taxa*

A total of 186 morphotypes were isolated, most (66 %) of which were Gram-negative. After screening for desirable probiotic activity, the most promising isolates were identified based on analyses of the 16S rRNA gene sequences. They belonged to the following genera: *Bacillus* (isolates 1, 50, 101, 108), *Enterobacter* (isolates 30, 116, 127), *Enterococcus* (isolates 131, 135) and *Marinomonas* (isolate 227) (Table 2; source raw sequence data can be downloaded from https://doi.org/10.5281/zenodo.6634279).

**Table 2.** Molecular identification of bacterial isolates of cobia culture with probiotic potential and their source of isolation

Isolate	Source	Identification
1	Water	* Bacillus* sp.
30	Water	* Enterobacter* sp.
50	Water	* Bacillus* sp.
101	Gill	* Bacillus* sp. 101
108	Intestine	* Mesobacillus boroniphilus *
116	Intestine	* Enterobacter* sp.
127	Rotifer	* Enterobacter cloacae *
131	Artemia	* Enterococcus devriesei *
135	Artemia	* Enterococcus casseliflavus *
227	Gill	* Marinomonas communis *

*Assays on activities in the bacterial isolates showing positive probiotic features*

Regarding antimicrobial activity of ECPs, 22 % of selected isolates had the ability to partially inhibit (>25 %) *A. hydrophila *, *V. alginolyticus *, *E. tarda* and *S. agalactiae* (Table 3). Isolates 227, 135, 131 and 127 inhibited more than 40 % of all the pathogenic bacteria evaluated (Table 4). Regarding QQ, 15 % of the isolates (live bacteria) generated an inhibition halo wider than 1 mm, whereas only 4 % of ECPs generated the same inhibition. Active bacterial isolates 1, 101, 116 and 135 inhibited violacein production and only in the case of isolate 127 did both ECPs and live active bacteria show QQ activity. Furthermore, ECPs of isolate 131 showed the ability to significantly reduce the biofilm formation of *Photobacterium* sp. and *B. pumillus* sp.

**Table 3.** Percentage inhibition of pathogenic bacteria incubated for 24 h with extracellular products from bacterial isolates with probiotic potential

ID	* A. hydrophila *	* S. agalactiae *	* E. tarda *	* V. alginolyticus *
1	0.0	8.3	9.9	24.5
30	50.5	18.6	26.7	18.6
50	0.0	0.5	0.0	60.2
101	27.8	26.8	39.6	49.7
108	29.9	23.2	30.6	48.7
116	0.0	31.4	30.3	56.2
127	47.9	46.8	64.7	52.1
131	46.0	50.4	62.1	53.4
135	43.5	54.3	58.8	55.8
227	50.4	48.7	53.3	55.7

**Table 4.** Inhibition of quorum sensing (QS) of bacterial isolates with probiotic potential Data are presented as the inhibition radius of violacein production in *C. violaceum* in millimetres.

Isolate	Live bacteria	ECPs
1	2	0
30	0	0
50	0	0
101	2	0
108	0	0
116	2	0
127	1	0.5
131	0	0
135	1.5	0
227	0	0

*Taxa of the most promising bacterial probiotic candidates isolated from cobia are correspondingly detected as key microbiome components of the healthy status*

**Fig. 3.**
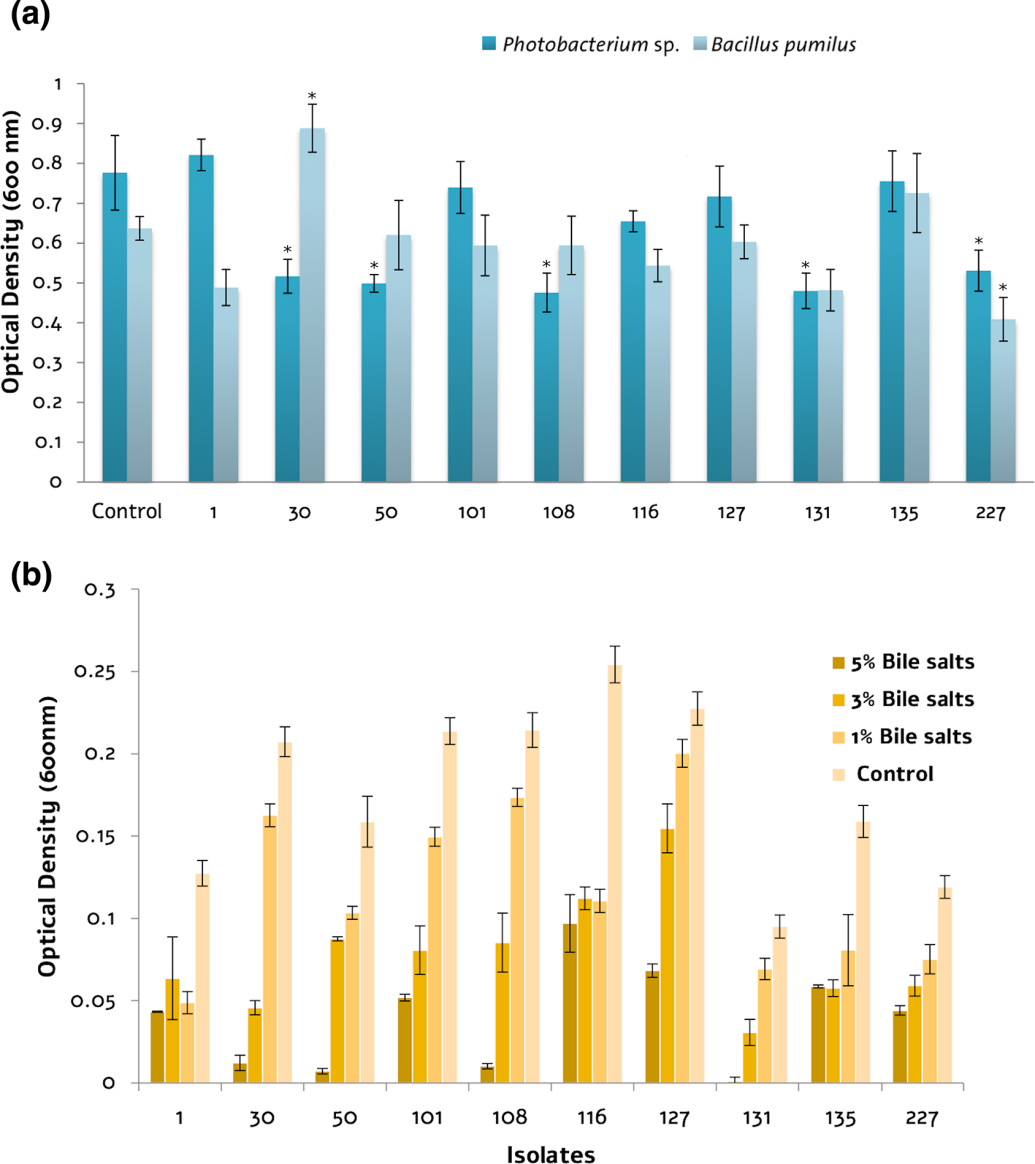
Inhibition of biofilm formation and survival with bile salts or isolates with probiotic potential recovered from cobia samples. (**a**) Activity of ECPs from selected bacteria with probiotic potential. Data are expressed as optical density ±se. An asterisk above the bar indicates a significant difference (*P*≤0.005) between each isolate and control. (**b**) Survival percentage of cobia culture isolates at different bile salts concentrations ±se. Bacterial growth at all bile salts concentrations was significantly lower than in controls (*P*≤0.05).

The *in vitro* characterization and identification of the ten isolates showing high probiotic potential is presented in Supplementary Table S1 (available in the online version of this article). Most isolates were sensitive to the antibiotics studied. Isolates 30, 101 and 108 were resistant to streptomycin, isolate 116 to ciprofloxacin, and isolate 227 to tetracycline. On the other hand, the other isolates studied were sensitive to all antibiotics tested (Supplementary Table S2). Assays performed with the API ZYM system revealed that isolates 108 and 127 had a higher enzymatic activity, mainly phosphatase activity. For lipases, none of the isolates reached an activity >20 nmol. The values for trypsin and chymotrypsin activity were also relatively low (< 20 nmol in all cases) except for isolate 127 with a value >30 nmol. Regarding survival in bile salts, isolates 1, 116, 127, and 227 had the ability to survive on bile salts. However, isolates 30, 50, 108 and 131 showed growth of less than 10 % in the treatment with 5 % bile salts (Fig. 3). Considering these results, ten isolates exhibited good composite features as potential probiotic strains. For the *Bacillus* spp., these are in particular agreement with its microbiome detection in healthy cycles.

## Discussion

Cobia culture extends across more than 15 countries from Oceania, Asia and America. Although protocols and techniques for larviculture have been contunually developed, final survival is still low mainly due to infectious diseases [1].

In 2013 we described an outbreak of epitheliocystis in cobia larvae from Colombian cultures. Its evaluation was carried out through a PCR-specific detection and *in situ* hybridization assays of a DIG-labelled PCR probe on the individuals with epitheliocysts signs. The results suggested that the aetiology could be attributed to a bacterial agent howing sequence similarity to *Endozoicomonas elysicola* [4]. In this work we performed detailed tracking of microbiome composition in cobia gill samples from healthy and diseased cohorts; although distinct patterns were found in the communities associated with both sick and healthy larvae in the cycles tracked, *Endozoicomonas* was not identified as a key player correlated with a health status or epitheliocystis. The DIG-labelled PCR probe was generated with primers specifically targeting an *E. elysicola* 16S rRNA gene fragment. The positive hybridization signal previously reported for cobia epithelyocystis could be produced either by bacterial strains closely related to *E. elysicola* abundantly present in the assay of that infection cycle or by a general detection of bacterial 16S rRNA gene sequences in the cysts. Under the conditions assayed, the labelled fragment could hybridize with 16S rRNA genes of bacteria belonging to a diverse range of different taxa. This 0.5 kb probe shows on average >83 % continuous 16S rRNA gene sequences similarity across its complete length to many type strains of Gram-positive and Gram-negative bacteria, such as *Bacillus* spp. or *Vibrio* spp. In this report we detected two OTUs classified as belonging to the genera *Bacillus* and *Vibrio* clearly associated with communities present in sick larvae, also characterized by a low relative abundance of the three OTUs that are, by contrast, the most abundant in the larva from healthy culture cycles with no apparent signs of disease. The OTUs associated with a healthy larval status were classified as belonging to the *Bacillus cereus* clade whereas the other two belong to unidentified genera of the family *Rhodobacteraceae *. The two samples from the healthy cycle that show a pattern closer to the diseased status (A04 and A06) (Fig. 2) represent two larvae that were probably at an early stage of disease development with no visual evidence yet. This culture-independent characterization indicates that both the bacterial types associated with health and disease are autochthonous with the microbiome content from the animal and the seawater.

*Vibrio* has been previously identified as a pathogen of cobia but produces skin ulcers as its main symptom [27], but our results suggest that this genus could, alone or synergistically, be also involved in the development of gills cysts in this fish. Regarding the *Bacillus* strains found in our study, the centroid representative sequence of the abundant OTUs was clearly distinct between health and disease conditions. The difference in the structure of the communities associated with affected and unaffected larvae, as described in this study, suggests that the appearance of cysts in the gills of cobia may be due not only to the presence of particular opportunistic or virulent bacterial types, such as the strains of *Vibrio* and *Bacillus* detected, but also to the decrease in the frequency of some specimens that might be refractory to disease, such as the OTU classified in the *Bacillus cereus* clade. This could be due to environmental stressors affecting the animals’ physiology and immune system. Strains of this bacterial species could be fundamental for the fish, due to the properties reported for some of them in producing toxins that could serve as antibacterial substances that prevent the colonization of pathogens [28].

Accordingly, we report here the isolation of potential bacterial probiotic strains associated to healthy cobia cultures. *Bacillus *, *Enterobacter* and *Marinomonas* were the most common genera found. After their biochemical characterization, those isolates were shown to have desirable activities for a bacterial probiotic such as QQ, the ability to survive in bile salts, and the presence of favourable enzymatic activity such as phosphatase, esterase lipase and leucine arylamidase, which could be involved in improved digestion of the host [29].

Regarding the *Bacillus* isolates, which were obtained from the intestine of healthy cobia larvae, antibacterial activity was observed in the ECPs by inhibition of pathogenic bacteria such as *A. hydrophila *, *E. tarda *, *V. alginolyticus* and *S. agalactiae *. Our results suggested good potential of isolate 108, the 16S rRNA gene sequence of which was closely related to the recently reclassified *Mesobacillus boroniphilus,* previously known as *Bacillus boroniphilus *. Strains of this species have been successfully used as probiotic in aquaculture systems, and their abilities to inhibit the growth of Gram-positive and Gram-negative bacteria [30] and the tolerance to high concentrations of boron [31] have been reported. Likewise, *Bacillus* sp. isolate 1 showed high QQ activity, which is related to interference in the expression of bacterial virulence genes such as toxic exoproteases, pathogenic traits that have been previously reported in various species of Vibrio [32]. Additionally, the isolated strains from the genera *Enterobacter* and *Marinomonas* also showed antibacterial activity, being able to reduce pathogenic bacterial growth from 48 to 55 %.

On the other hand, regarding the infectious nature of the epitheliocystis, here we provide additional support for the bacterial aetiology as inferred from the first analyses of the outbreak. *Endozoicomonas* spp. was initially proposed as the possible aetiological agent [4]. This work in new cobia cohorts tracked across growth stages, with extensive sampling, and throughout an in-depth molecular detection, indeed provides evidence for dysbiosis in the microbiome of sick larvae, where strains belonging to *Vibrio* spp. and *Bacillus* spp. could play a key aetiological role. Risk factors for disease development could be caused by a combination of environmental and holobiont variables that have been reported to be of pivotal importance for cobia aquaculture [33, 34]. Lower surface water temperature and harsh disinfectant regimes could damage protective animal tissues, causing the refraction of pathogens to the symbiotic microbiome, which originally consists mainly of a non-pathogenic and possibly probiotic *Bacillus* sp. ecotype similar or identical to strain 101 recovered, and to as yet uncultured *Rhodobactereacea* ecotypes. This alteration is followed by colonization of a proinflammatory pathobiont synergistic community composed mainly of distinct *Bacillus* and *Vibrio* strains.

Note that taxa that have been previously identified as bacterial pathogens of cobia, such as *Chlamydia* [35], *Aeromonas* [36], *Mycobacterium* [37] and *Photobacterium* [38], were not detected as predominant members of the microbiomes in the samples analysed here. However, we did frequently recover from sick cycles bacterial isolates belonging to *Vibrio* and *Photobacterium* species (see Supplementary Table S1). It is noteworthy that despite the well-known limitations for specific intergenus and intraspecies discrimination of *Mesobacillus* and *Bacillus* species by 16S rRNA gene sequencing, in this study it was possible to differentiate among the OTUs classified in these genera in both healthy and diseased cycles, associating those related to the probiotic isolates. Following our results, gene families with better discriminatory power could be used for cases such as this, where *Mesobacillus–Bacillus* are already identified as key members of the probiosis–dysbiosis balance.

The main conclusions from our study are based on a relatively large number of samples, tracking the growth cycles of health and disease with a high-resolution culture-independent technique, and the detection of probiotic potential in bacterial isolates. Note that there many new open questions that may need additional techniques and samples to confirm and refine our current assessments: i.e. shotgun genomics of the isolates or metagenomics from cobia tissues could provide complementary information on microbial genes and their associated functions and taxa. Besides, tracking more rearing cycles would increase the representation of biological data for healthy and diseased cycles; detailed tracking of more physical chemical parameters, extensive histopathological analyses, and specific probes of specimens at different stages together with infection challenge assays of potential pathogens recovered from cobia, and probiotic performance under controlled conditions *in vivo* are certainly research paths worthy of exploration in further studies. They may provide additional evidence for a solid understanding of the microbes underlying epitheliocystis of cobia.

To summarize, our results suggest that epithelocystis in cobia seems to be favoured by a combination of environmental and biological factors, followed by an increase of colonizing pathobionts inducing or exacerbating stress, inflammation and cytological abnormalities. As a general application using this model, this study shows the feasibility of using microbiome information to direct efforts towards probiotic strain isolation. This additional assessment adds a layer of information about the autochthonous and ecological nature of the strains collected that have probiotic apotential.

## Supplementary Data

Supplementary material 1
